# Resilience, successful aging, and hope in hospitalized older people

**DOI:** 10.15649/cuidarte.4933

**Published:** 2025-09-01

**Authors:** Víctor Alfonso Benítez Rodríguez, Tirso Duran-Badillo, Jesus Alejandro Guerra Ordoñez, Xochitl Pérez Zúñiga, Luis Carlos Cortez González

**Affiliations:** 1 Universidad Autónoma de Tamaulipas, Unidad Académica Multidisciplinaria Matamoros, Heroica Matamoros, Tamaulipas, México. E-mail: victor.benitez@uat.edu.mx Universidad Autónoma de Tamaulipas, Unidad Académica Multidisciplinaria Matamoros Tamaulipas México victor.benitez@uat.edu.mx; 2 Universidad Autónoma de Tamaulipas, Unidad Académica Multidisciplinaria Matamoros, Heroica Matamoros, Tamaulipas, México. E-mail: tduran@docentes.uat.edu.mx Universidad Autónoma de Tamaulipas, Unidad Académica Multidisciplinaria Matamoros Tamaulipas México tduran@docentes.uat.edu.mx; 3 Universidad Autónoma de Tamaulipas, Unidad Académica Multidisciplinaria Matamoros, Heroica Matamoros, Tamaulipas, México. E-mail: jesus.guerra@docentes.uat.edu.mx Universidad Autónoma de Tamaulipas, Unidad Académica Multidisciplinaria Matamoros Tamaulipas México jesus.guerra@docentes.uat.edu.mx; 4 Universidad Autónoma de Tamaulipas, Unidad Académica Multidisciplinaria Matamoros, Heroica Matamoros, Tamaulipas, México. E-mail: xperez@docentes.uat.edu.mx Universidad Autónoma de Tamaulipas, Unidad Académica Multidisciplinaria Matamoros Tamaulipas México xperez@docentes.uat.edu.mx; 5 Universidad Autónoma de Coahuila, Facultad de Enfermería, Saltillo, Coahuila, México. E-mail: lucortezg@uadec.edu.mx Universidad Autónoma de Coahuila, Facultad de Enfermería Coahuila México lucortezg@uadec.edu.mx

**Keywords:** Resilience, Psychological, Healthy Aging, Hope, Aged, Hospitalization, Resiliencia Psicológica, Envejecimiento Saludable, Esperanza, Anciano, Hospitalización, Resiliência Psicológica, Envelhecimento Saudável, Esperança, Idoso, Hospitalização

## Abstract

**Introduction:**

The global population aging and the rise in chronic diseases pose challenges in the care of hospitalized older adults. Factors such as hope and resilience may influence their recovery.

**Objective:**

To determine the relationship between resilience and successful aging with hope in hospitalized older adults.

**Materials and Methods:**

A correlational-predictive study was conducted with 385 hospitalized older adults in Matamoros, Tamaulipas, Mexico. Validated instruments were used to measure hope, resilience, and successful aging. Data were collected by the principal investigator across all work shifts during the first half of 2024.

**Results:**

The mean age of participants was 70.65 ± 6.11 years, and 53.2% were women. The lesding causes of hospitalization were surgery (42.3%), acute illness (28.8%), and chronic disease (18.7%). Mean scores of resilience (76.34 ± 21.61), successful aging (60.76 ± 16.99), and hope (36.27 ± 6.64) were obtained. Resilience and successful aging were significant predictors of hope, accounting for 15% of its variance (p < 0.05).

**Discussion:**

The relationships found align with findings from other research.

**Conclusion:**

The findings suggest that strengthening resilience and promoting successful aging may help sustain hope in hospitalized older adults, providing a foundation for future clinical interventions.

## Resumen

**Introducción:**

El envejecimiento poblacional global y el incremento de enfermedades crónicas generan desafíos en la atención de la persona mayor hospitalizada. Factores como la esperanza y la resiliencia pueden influir en su recuperación.

**Objetivo:**

Determinar la relación entre la resiliencia y el envejecimiento exitoso con la esperanza de las personas mayores hospitalizadas.

**Materiales y métodos:**

Se realizó un estudio correlacional-predictivo en 385 personas mayores hospitalizados en Matamoros, Tamaulipas, México. Se aplicaron instrumentos validados para medir esperanza, resiliencia y envejecimiento exitoso. La información se obtuvo en el primer semestre del 2024 por el investigador principal, en todos los turnos.

**Resultados:**

La muestra presentó una edad promedio de 70,65 ± 6,11 años, el 53,2 % eran mujeres y los principales motivos de hospitalización fueron cirugía (42,3 %), enfermedad aguda (28,8 %) y enfermedad crónica (18,7 %). Se encontraron puntuaciones promedio de resiliencia (76,34 ± 21,61), envejecimiento exitoso (60,76 ± 16,99) y esperanza (36,27 ± 6,64). La resiliencia y el envejecimiento exitoso fueron predictores significativos de la esperanza, explicando el 15 % de su varianza (p <0,05).

**Discusión:**

Las relaciones encontradas son congruentes con lo reportado en otras investigaciones.

**Conclusiones:**

Los hallazgos sugieren que fortalecer la resiliencia y promover un envejecimiento exitoso puede contribuir a mantener la esperanza en personas mayores hospitalizadas, ofreciendo una base para futuras intervenciones en el ámbito clínico.

## Resumo

**Introdução:**

O envelhecimento populacional global e o aumento de doenças crônicas representam desafios no cuidado de idosos hospitalizados. Fatores como esperança e resiliência podem influenciar sua recuperação.

**Objetivo:**

Determinar a relação entre resiliência e envelhecimento bem-sucedido e a esperança de idosos hospitalizados.

**Materiais e métodos:**

Um estudo correlacional-preditivo foi conduzido com 385 idosos hospitalizados em Matamoros, Tamaulipas, no México. Instrumentos validados foram utilizados para mensurar esperança, resiliência e envelhecimento bem-sucedido. Os dados foram coletados durante o primeiro semestre de 2024 pelo pesquisador principal, em todos os plantões.

**Resultados:**

A amostra apresentou média de idade de 70,65 ± 6,11 anos, 53,2% eram mulheres e os principais motivos de hospitalização foram cirurgia (42,3%), doença aguda (28,8%) e doença crônica (18,7%). Foram encontradas pontuações médias para resiliência (76,34 ± 21,61), envelhecimento bem-sucedido (60,76 ± 16,99) e esperança (36,27 ± 6,64). Resiliência e envelhecimento bem-sucedido foram preditores significativos de esperança, explicando 15% de sua variância (p < 0,05).

**Discussão:**

As relações encontradas são consistentes com aquelas relatadas em outras pesquisas.

**Conclusões:**

Os resultados sugerem que o fortalecimento da resiliência e a promoção do envelhecimento bem-sucedido podem contribuir para a manutenção da esperança em idosos hospitalizados, oferecendo uma base para futuras intervenções no ambiente clínico.

## Introduction

Population aging has been steadily increasing. Global population projections indicate that by 2050, 22% of the world's population will be older adults, and 80% residing in low- and middle-income countries. Therefore, all countries are being urged to prepare their health systems to meet the emerging needs associated with these demographic shifts^1^. Mexico is no exception: its older adult population is growing, along with life expectancy, which is expected to approach 80 years by 2050^2^.

The increase in both the number of older adults and in life expectancy brings with it the need to focus attention on the well-being of this population group^3^. However, the increase in chronic diseases and associated risk factors^4^, as well as polypharmacy^5^, elevates the risk of hospitalization. When faced with hospitalization, it is not only physical healthcare that is important, but also mental healthcare for the hospitalized individuals. It is at this critical juncture that hope becomes important and can serve as a protective factor for mental health and therapeutic prognosis^6^.

Hope is a feeling, expectation, or positive attitude toward something that is desired, which motivates individuals to take positive actions to achieve it^7^. Accordingly, it is suggested that addressing hope in hospitalized older adults may contribute to treatment adherence, which in turn can lead to reduced emergency visits, hospital admissions, and mortality^8^.

The positive effect of hope on hospitalization in older adults justifies the need to identify its predictive variables. One such variable may be resilience, understood as a psychological resource that helps individuals cope with stressful events, such as hospitalization, by promoting adaptive capacity^9^.

In a review of the literature, little evidence has been found of studies addressing resilience in older adults. Reported mean resilience scores among this population range from 26 to 78 points^10^-^12^. For hospitalized older adults, resilience levels have been reported to be 90% within the medium to high range^9^. However, no studies have been identified that examine the relationship between resilience and hope.

Another variable that could predict hope is successful aging. Garófalo^13^ and Torregrosa-Ruiz^14^ suggest that successful aging is a combination of physical, mental, and social components. Although these components can be affected by aging, their significance lies in how older individuals cope with these changes. It has been reported that hospitalized older adults have more frequently chronic diseases, malnutrition, physical decline, depressive symptoms, and reduced engagement in work and social activity; these situations suggest a lower likelihood in this population of experiencing successful aging^15^.

Considering the health conditions of hospitalized individuals and the negative impact these conditions have on perceptions of successful aging^15^, it is reasonable to argue that hope may be adversely affected, which could, in turn, negatively impact the individual's recovery process. However, this remains a premise, as no studies have yet explained the relationship between successful aging and hope in hospitalized older adults.

For gerontological nurses, it is essential to identify the factors that predict or explain hope, since considering hope as a protective factor against adverse events, such as hospitalization, will enable the development of nursing interventions that contribute to strengthening hope during hospitalization, thereby impacting coping and facilitating a speedy recovery. This research aimed to investigate the relationship between resilience and successful aging in hospitalized older adults and their hope.

## Materials and Methods

A correlational-predictive study was conducted on a population of older adults hospitalized at a public hospital in Matamoros, Tamaulipas, Mexico. As the number of hospitalized patients was unknown, the sample size was calculated for an infinite population, assuming an absolute precision of 0.05, a 95% confidence level, and a 50% probability of the problem occurring. The sample size was, therefore, 385 hospitalized older people. The study subjects were selected through convenience sampling. The primary inclusion criterion was being an older adult hospitalized in the internal medicine, surgery, or trauma departments. Additionally, participants were required to have been hospitalized for at least three days. To verify the person, time, and place criteria, the patient's name, date of admission, and hospital name were confirmed.

A personal data form was used to collected information on age, sex, marital status, educational level, cohabiting family members, religion, diagnosed chronic conditions, and prescribed and non-prescribed medications. The date, reason for hospitalization, and the department of admission, were obtained from patients' medical records.

The Herth Hope Index (HHI)^16^ includes 12 items rated on a Likert scale from "strongly agree" (4) to "strongly disagree" (1). Items 3 and 6 are reverse-scored due to their negative phrasing. The total score ranges from 12 to 48 points, with higher scores indicating greater levels of hope. The HHI was validated in the Peruvian population by Castilla-Cabello et al.^17^, who reported a Cronbach's alpha of 0.85 and satisfactory reliability in the Spanish population of ω = 0.88^18^. This version has been used on older adults in Mexico^19^.

The Connor-Davidson Resilience Scale (CD-RISC)^20^ consists of 25 items rated on a Likert-type scale from 0 (strongly disagree), 1 (not at all), 2 (rarely), 3 (sometimes), to 4 (almost always). Scores range from 0 to 100, with higher scores indicating greater resilience. The instrument was translated into Spanish and demonstrated a Cronbach’s alpha of 0.86^21^.

The Successful Aging Inventory (SAI), developed by Troutman et al.^22^, contains 20 items rated on a Likert scale from 0 (strongly disagree) to 4 (strongly agree). Considering that higher scores indicate more successful aging, scores between 0 and 25 indicate unsuccessful aging, between 26 and 53 indicate moderately successful aging, and between 54 and 80 indicate successful aging^23^. The SAI was translated into Spanish and validated for use in the Mexican population, with a Cronbach's alpha of 0.85^24^.

The research protocol was reviewed and approved by a research and ethics committee (Decision 115). Approval was obtained from hospital authorities, and informed consent was provided by all the patients. Fieldwork was conducted by the principal investigator in accordance with the ethical principles for research^25^. The invitation and administration of instruments were conducted individually in each patient's bed. Data was collected during the first half of 2024 by the principal investigator and covered all hospital shifts.

Data were processed and analyzed using the Statistical Package for the Social Sciences (SPSS), version 21.0 for Windows. Descriptive statistics were used to summarize participant characteristics and study variables. The Kolmogorov-Smirnov test with Lilliefors correction was applied. Since the variables did not follow a normal distribution, non-parametric statistics were used. A univariate linear regression model was then developed, with hope as the dependent variable and resilience and successful aging as the independent variables. The results were considered statistically significant when the p-value was equal to or less than 0.05. The dataset is available in the Mendeley Data repository^26^.

## Results

A total of 53.20% (f=205) of participants were female. Additionally, 51.40% (f=198) reported not having a partner, 78.20% (f=301) identified as practicing a religion, 70.90% (f=273) reported having a chronic illness, and 42.30% (f=163) had been hospitalized for surgery (Table 1). Regarding the length of hospitalization, patients had a minimum stay of 3 days and a maximum of 71 days (5.92 ± 6.252 days). The average age was 70.65 years (± 6.11), and the mean number of years of formal education was 6.81 (± 3.697) years. 

**Table 1:** Characteristics of older adults

Variables	% (f)
Sex	
Female	53.20 (205)
Male	46.80 (180)
Marital status	
With a partner	48.60 (187)
Without a partner	51.40 (198)
Practices religion	
Yes	78.20 (301)
No	21.80 (84)
Chronic disease diagnosis	
Yes	70.90 (273)
No	29.10 (112)
Reason for hospitalization	
Chronic disease	18.70 (72)
Acute disease	28.80 (111)
Surgery	42.30 (163)
Psychiatric disease	3.40 (13)
Accident	6.80 (26)

f = frequency

 The following mean scores were observed: resilience (76.34 ± 21.609) successful aging (60.76 ± 16.991) and hope (36.27 ± 6.635) (Table 2). Regarding levels of successful aging, 70.60% (f=272) demonstrated successful aging, 25.50% (f=98) were classified as moderately successful, and 3.90% (f=15) exhibited unsuccessful aging. 

**Table 2:** Descriptive statistics for resilience, successful aging, and hope among hospitalized older adults

Variables	Min	Max	Mean ± SD
Resilience	2	100	76.34 ± 21.609
Successful aging	0	80	60.76 ± 16.991
Hope	15	47	36.27 ± 6.635

SD= Standard deviation

A positive and statistically significant relationship was found between resilience (rs = 0.519; p<0.001) and successful aging (rs = 0.539; p<0.001) and hope. Subsequently, a univariate linear regression analysis was conducted, with resilience and successful aging entered as independent variables and hope as the dependent variable. The predictive effect of resilience and successful aging was found to be statistically significant (F[2, 382] = 35.449; p<0.001; R2= 0.157), allowing us to conclude that these two variables together explain 15.00% of the variance in hope among hospitalized older adults (Table 3). 

**Table 3:** General linear model for resilience, successful aging, and hope among hospitalized older adults

Variables	β	95% CI		p-value
		LL	UL	
**R^2^ = 0.157**				
Corrected model				< 0.001
Intercept	26.267	23.83	28.69	< 0.001
Resilience	0.080	0.03	0.12	< 0.001
Successful aging	0.065	0.01	0.11	0.015

β = Beta regression coefficient; p-value = statistical significance; LL= Lower limit; UL= Upper limit.

## Discussion

This research found that both resilience and successful aging are predictors of hope. The relationship observed between resilience and hope is similar to that reported by other authors^27^. This suggests that, when faced with challenging situations such as hospitalization, older adults with protective psychological resources are better able to adapt, enabling them to cope with the process more calmly^11^. Additionally, the study found that the more successful the aging process was, the greater the hope among hospitalized older adults, a finding that aligns with recent findings by Troutman et al.^22^. This suggests that successful aging helps maintain calmness and objectivity during hospitalization.

These findings support the importance of implementing nursing interventions to strengthen resilience and promote successful aging from early stages of life. By doing so, older adults will have internal resources that help them to sustain hope when hospital care is needed. The mean resilience score found in this study was lower than that reported in other studies involving Mexican older adults who live with family members and do not disclose health conditions^10^. In contrast, the older adults in this study were hospitalized, approximately half reported not having a partner, and nearly three-quarters reported suffering from at least one chronic disease. This difference may be explained by the fact that self-perceived health, economic status, and support networks are factors associated with resilience^28^.

The successful aging score reported in this study is higher than those found in studies conducted in China^29^ and Colombia^30^. However, higher scores were reported in a Chilean study. These differences may be attributable to the context of the study conducted by Gallardo Peralta et al.^23^, which focused on an indigenous population that maintains its cultural practices, an aspect that could impact perceptions and ways of aging. Given that the present study was conducted in a border city with residents originating from various regions of Mexico, this cultural diversity suggests the need for future research to explore participants' places of origin and the duration of their residence in this city.

Regarding hope, the average score observed was similar to that reported by Silveira et al.^31^ and showed a tendency toward the results of Silva et al.^32^, both of which were conducted in Brazil. However, the findings reported by Ortiz et al.^11^ in a study of older adults with chronic conditions in Colombia were higher than those found here. In the present study, only three-quarters of participants reported having a chronic disease. This result may suggest that hope becomes stronger when individuals are confronted with chronic disease, possibly because they learn to live with their condition.

Among the strengths of this study is its correlational-predictive approach, which enabled the exploration of significant relationships between relevant psychosocial variables within the clinical context of hospitalized older adults. Additionally, the use of validated instruments and a sufficiently large sample size for the statistical analyses conducted is noteworthy. However, some limitations should be acknowledged. The cross-sectional design prevents the establishment of causal relationships between the variables analyzed. Moreover, the use of non-probability convenience sampling limits the generalizability of the results to other populations or contexts. Another limitation is the exclusion of potentially influential variables, such as perceived social support, functional status, or cognitive functioning, which may play a relevant role in the experience of hope during hospitalization. It is recommended that future research adopt longitudinal designs and consider a broader range of biopsychosocial variables to enhance the understanding of this phenomenon.

Overall, the findings of this study emphasize that future research within hospital settings should consider strategies to promote resilience and successful aging to have a positive impact on the hope of hospitalized older adults. Among the interventions that could be used are assessing available resources for goal attainment, conducting objective assessments of events, adopting constructive problem solving strategies, enhancing support systems, fostering hope, reducing anxiety, supporting grief processing, promoting self-responsibility, facilitating the expression of guilt, identifying risks, implementing crisis interventions, improving coping, providing anticipatory guidance, using relaxation techniques and therapies, boosting self-confidence, and facilitating integration into support groups^33^. These interventions can help strengthen hope in older adults, with an impact on their physical and/or emotional health.

## Conclusions

This research identified resilience and successful aging as protective factors associated with hope in hospitalized older adults. These findings underscore the need for nursing professionals to implement interventions that target these protective variables, as they are considered important for promoting physical and mental health, and ultimately, enhancing the quality of life in this vulnerable population.

Future research should consider including variables such as city of origin and length of time since migration, as well as other factors related to resilience, to positively impact successful aging. Moreover, it is necessary to propose experimental studies aimed at fostering resilience in older adults, which will have a positive impact on successful aging.
